# Species turnover drives *β*-diversity patterns across multiple spatial scales of plant-galling interactions in mountaintop grasslands

**DOI:** 10.1371/journal.pone.0195565

**Published:** 2018-05-18

**Authors:** Marcel Serra Coelho, Marco Antônio Alves Carneiro, Cristina Alves Branco, Rafael Augusto Xavier Borges, Geraldo Wilson Fernandes

**Affiliations:** 1 Ecologia Evolutiva & Biodiversidade, DBG, ICB, Universidade Federal de Minas Gerais, Belo Horizonte, Minas Gerais, Brazil; 2 Laboratory of Phenology, Department of Botany, IB UNESP, UNESP Universidade Estadual Paulista, Rio Claro, São Paulo, Brazil; 3 Laboratório de Entomologia Ecológica/DEBIO, ICEB, Universidade Federal de Ouro Preto, Ouro Preto, Minas Gerais, Brazil; 4 Laboratorio de Botânica, Universidade Federal do Rio Grande do Sul, Porto Alegre, Rio Grande do Sul, Brasil; Chinese Academy of Forestry, CHINA

## Abstract

This study describes differences in species richness and composition of the assemblages of galling insects and their host plants at different spatial scales. Sampling was conducted along altitudinal gradients composed of *campos rupestres* and *campos de altitude* of two mountain complexes in southeastern Brazil: Espinhaço Range and Mantiqueira Range. The following hypotheses were tested: i) local and regional richness of host plants and galling insects are positively correlated; ii) beta diversity is the most important component of regional diversity of host plants and galling insects; and iii) Turnover is the main mechanism driving beta diversity of both host plants and galling insects. Local richness of galling insects and host plants increased with increasing regional richness of species, suggesting a pattern of unsaturated communities. The additive partition of regional richness (γ) into local and beta components shows that local richnesses (α) of species of galling insects and host plants are low relative to regional richness; the beta (β) component incorporates most of the regional richness. The multi-scale analysis of additive partitioning showed similar patterns for galling insects and host plants with the local component (α) incorporated a small part of regional richness. Beta diversity of galling insects and host plants were mainly the result of turnover, with little contribution from nesting. Although the species composition of galling insects and host plant species varied among sample sites, mountains and even mountain ranges, local richness remained relatively low. In this way, the addition of local habitats with different landscapes substantially affects regional richness. Each mountain contributes fundamentally to the composition of regional diversity of galling insects and host plants, and so the design of future conservation strategies should incorporate multiple scales.

## Introduction

Galling insects are among the most specialized and fascinating herbivores due to their ability to control host plant development, and thus are considered one of the most sophisticated herbivores [1]. Plant galls or tumors are cells, tissues or plant organs formed by abnormal symmetric growth in response to an increase in the number (hyperplasia) and volume (hypertrophy) of cells in response to feeding or other stimuli from an invading organism [2,3,4,5]. From an evolutionary point of view, galls can be seen as extended insect phenotypes [6] or adaptations of their inductors for feeding on a high quality tissue and for protection from environmental variation and natural enemies [7,8,9,10].

Gall-inducing insects are more species rich and abundant in xeric habitats (high temperatures, low humidity and nutrient-poor soils) than in mesic habitats (low temperatures, high humidity and nutrient rich soils) [11,12,13]. Previous work has shown that the relationship between species richness of galling insects and latitude/altitude is a result of increased hygrothermal and nutritional stresses of habitats in intermediate latitudes and low altitudes, respectively [13]. The mechanisms responsible for the occurrence of more species in xeric habitats are habitat-mediated survival and mortality [12,14]. On the other hand, [15] observed that fire is a common phenomenon in habitats with sclerophyllous vegetation and could be an important selective force on the distribution of galling insects. In these environments, the insects would benefit because fire would promote the synchronization of the production of new vigorous branches for colonization. The mechanism responsible for the occurrence of more species in xeric habitats is the increase in oviposition errors due to the synchronization of new branch growth after fire [15]. Fire was later included in the initial hypotheses of [11].

In addition to hygrothermal and nutritional stress, species richness and taxonomic composition of vegetation play important roles in the richness of galling insect species. Whereas [16,17] have shown that the richness of host plant species in *Fynbos* (South Africa), vegetation is the main determinant of species richness of galling insects; other studies considered that host plant richness is a poor indicator of the variation in species richness of galling insects in campos rupestres and in the Australian savanna [18,19,20].

Along altitudinal gradients, abiotic variation occurs with increasing ultraviolet radiation, decreasing temperature and increasing humidity with increasing altitude [21]. This environmental variation, combined with others (i.e., soil, topography), is accompanied by changes in the structure of communities, especially the number and composition of species. Therefore, mountains are excellent systems for testing ecological hypotheses [22,23]. One way to understand patterns of species richness along environmental gradients (i.e., altitude) is to separate richness into its different components. Total richness of a mountain, also called regional or gamma richness (D_gamma_) can be separated in to two components: the diversity of a local habitat, called alpha diversity (D_alpha_), which is the component that represents the average number of species occurring in a sample unit (habitat); and diversity among local communities, called beta diversity (Dbeta), which is the component that represents the difference in species composition between sample units or between habitats [24,25]. Beta diversity can be analyzed by both a multiplicative model (beta = gamma/alpha) and an additive model (beta = gamma-alpha), depending on the objectives and statistical design adopted [26,27,28]. In addition, beta diversity can be driven by two distinct phenomena: nesting and turnover. Nesting occurs when habitats with low richness host part of the species of richer habitats, which reflects a non-random process of disaggregation of assemblages. Turnover is a process of substitution of species by environmental selection or historical and spatial restriction [29]. Thus, although habitats in a landscape can be organized into different configurations they are all directed only by these two processes, or a combination thereof, because the only ways that distinct patterns can be generated in assemblages is by replacing or gaining species [28,29]. The understanding of these patterns of spatial distribution of species is of great relevance for designing conservation strategies because it can direct efforts to priority areas. Some studies have already demonstrated the importance of beta diversity for the regional diversity of galling insects, although there is still no record of the role played by both the mechanisms of turnover and nesting [30,22].

In general, it is accepted that herbivorous insect communities are locally unsaturated [31, 32, 33, 34]. This pattern suggests that local richness is independent of the antagonistic interactions that occur in the habitat; local richness is a proportional sample of regional richness [35,32,36,34,22]. Galling insect communities, as well as other herbivorous insect types are locally unsaturated, that is, local and regional richness are positively related [31,33,35]. Wasps (Cynipidae) that induce galls on species of the genus *Quercus*, for example, showed a positive relationship between local and regional species richness [32]. The same pattern of unsaturation was found for the community of galling insects on species of *Ficus* [36]. This pattern seems to follow those of unsaturation in host plant communities [22].

In this work, differences in species richness and composition of the assemblages of galling insects, as well as their host plants, are described for different spatial scales. Sampling was conducted along altitudinal gradients composed of rupestrian grasslands (Locally called campos rupestres) and altitudinal fields (locally called campos de altitude) of two mountain complexes in southeastern Brazil: Espinaço Mountain Range (Locally called *Cadeia do Espinhaço*) and Mantiqueria Mountain Range (Locally called *Serra da Mantiqueira*). The following hypotheses were tested: i) local and regional richness of plant hosts and galling insects are positively correlated; ii) beta diversity is the most important component of the regional diversity of the host plants and galling insects’ diversity; and iii) turnover is the principle mechanism driving the beta diversity of both host plants and galling insects.

## Methodology

### Study area

The open grasslands at high altitudes in southeastern Brazil were named “alpine fields” (campos alpinos) by [37] and “alti-montane fields” (campos altimontanos) by [38]. However, these classifications include ecosystems that, although they are physiognomically similar, differ in terms of lithological characteristics, geological origins and environmental matrices, as well as biological characteristics, such as floristic composition, for example. Due to such differences, fields located at high elevations were subdivided into “quartzitic” and “alti-montane” fields by [38] and later into “rupestrian grasslands” (campos rupestres) and “altitudinal fields” (campos de altitude) by [39].

Sampling was concentrated in regions of rupestrian grasslands in the Espinhaço Range (MG, BA) and altitudinal fields in the Mantiqueira Range (MG, RJ). The Espinhaço Range comprises a group of mountains between 20°35'S and 11°11'S, from the Serra de Ouro Branco, south of the city of Ouro Preto, in Minas Gerais, to Bahia, where it is called Chapada Diamantina [40]. Formed by intermittent uplifts beginning in the Paleozoic, the soils are shallow, sandy and poor in nutrients. The Espinhaço Range possesses the Cwb climate type of Köppen (mesothermic climate with mild summers and a rainy season in the summer), with average temperatures between 17.4° and 19.8°C, and an average temperature of the hottest month being below 22°C [41]. The annual precipitation of the region is around 1,500 mm, with a dry winter of 3 to 4 months, and a wet period of 7 to 8 months.

The formation of the Mantiqueira Range dates from the Cretaceous period, and comprises a rocky massif with a large upland area between 1,000 and almost 3,000 meters in altitude along the borders of the states of São Paulo, Rio de Janeiro and Minas Gerais. The largest portion of it is in Minas Gerais and is included within the Atlatnic Forest domain [42]. The region is under the influence of two Köppen climatic zones. Forest areas are classified as mesothermal Cfb, with annual temperatures ranging from 12°C to 20°C. The regions of grasslands and plateaus are classified Cwb, with average annual temperatures below 12°C, and annual precipitation varying from 1,500 to 2,000 mm in the altitudinal fields of Minas Gerias and São Paulo and can reach 3,000 mm in those of Rio de Janeiro [42,43].

**Fig 1:**
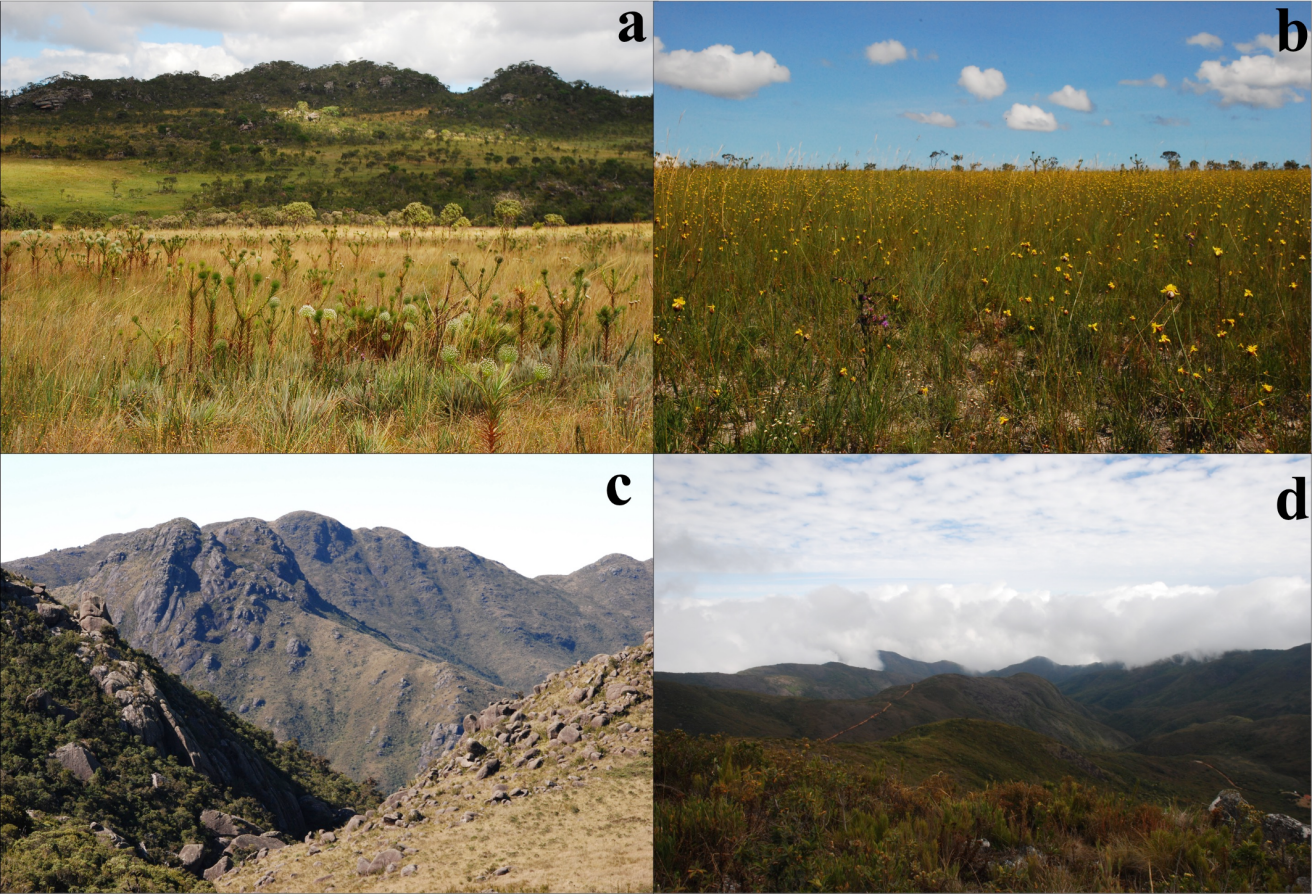
Photos of rupestrian grasslands and altitudinal fields sampled in this work. Photos “a” and “b” are of rupestrian grasslands in P.E. Serra do Cabral. Photos “c” and “d” are of de altitudinal fields in P.N. Itatiaia.

Rupestrian grasslands are predominantly formed by rocks such as quartzites and sandstones and are predominantly associated with the Cerrado domain, although they may occur immersed in matrices of other ecoregions [44]. Altitudinal fields occur predominantly on granite rocks within a matrix of Atlantic Forest, and are considered the Paramos of Brazil [45,46]. The vegetation of both physiognomies is predominately dominated by herbs and shrubs belong to Asteraceae, Melastomataceae, Orchidaceae, Bromeliaceae the most common families in the altitudinal fields and Asteraceae, Xyridaceae, Velloziaceae, Cyperaceae and Melastomataceae which are the most common botanical families in the rupestrian grasslands [47] (Fig 1). Altitudinal fields and rupestrian grasslands are priority areas for conservation because they provide important environmental services to Brazilian society, including stocking biodiversity and serving as a water supply [42,43,46,48, 49,50].

### Sample design

**Fig 2:**
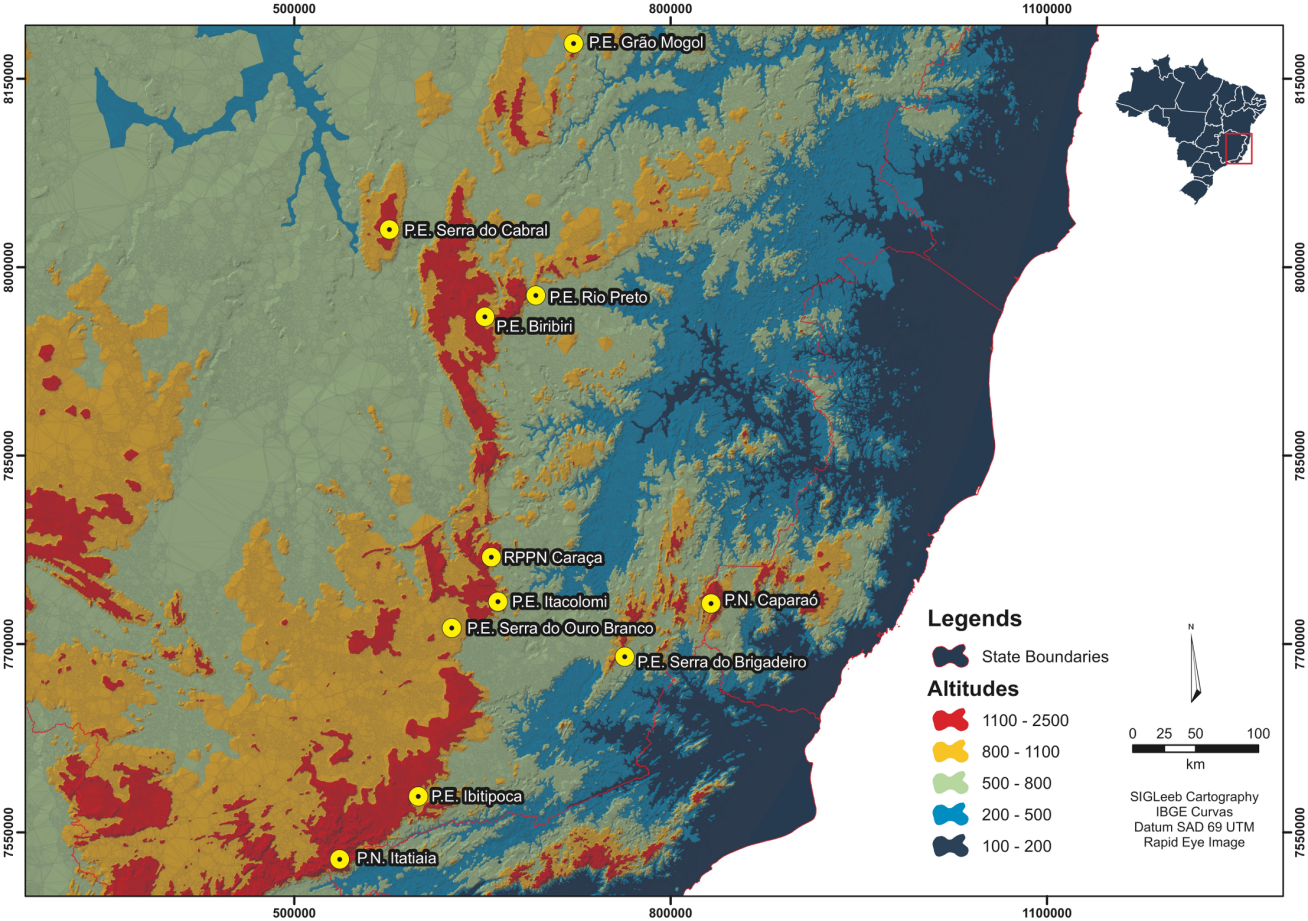
Hyposometric map with the location of the elevensampled mountains, four of which are associated with the Mantiqueira Range — P.N.Itatiaia, P.E.Ibitipoca, P.E.Serra do Brigadeiro and P.N. Caparaó — and seven associates with the Espinhaço Range — P.E. Serra do Ouro Branco, P.E. Itacolomi, RPPN Caraça, P.E. Biribiri, P.E. Rio Preto, P.E. Serra do Cabral and P.E. Grão Mogol.

For this project, 11 mountains were sampled including seven in the Espinhaço Range (P.E. Grão Mogol, P.E. Serra do Cabral, P.E. Biribiri, P.E. Rio Preto, RPPN Caraça, P.E. Serra do Ouro Branco and P.E. Itacolomi) and four in the Mantiqueira Range (PARNA do Itatiaia, Parque Estadual do Ibitipoca, MG, and Parque Estadual da Serra do Brigadeiro, PARNA do Caparaó) S1 File (Fig 2). The mountains were selected in order to maximize sampling along the latitudinal gradient and due to logistical facilities provided by the conservation units in these locations [22,51,52,53].

Sampling was performed following the methodology proposed by [18]. Ten sampling points of herbaceous-shrub vegetation were chosen at different altitudes. Areas of forest, areas close to trails and areas with any visible anthropogenic interference were excluded from sampling. For standardization, the gull richness of the first one-hundred woody herbaceous-shrub plants (up to 2 m high) were sampled by direct counting on the crown, for a total of 1000 individual plants per mountain and 11,000 plants overall for the study S1 File. Previous studies have demonstrated differences in the richness of gall morphotypes in vegetation of different architectures, focusing on the architecture of the plant as one of the explanatory factors for richness of gall inducing insects, with shrubs being the type of vegetation in which the greatest richness of galls is concentrated [54,55,56].

The description of gall morphotypes associated with host-plant species identification is a reliable indicator of the richness of gall-inducing insects [57]. About 95% of the species of cecidomyids described in Brazil can be identified based on their external form associated with the host plant in which it occurs, reinforcing this as a reliable methodology for use in studies with galling insects [13,20,58,59,60].

Galls and host plants were collected and brought to the laboratory for further analysis. All plants and galls were mounted, identified and deposited in the Herbarium BHCB of the Departamento de Botânica of the Universidade Federal de Minas Gerais. The classification of the host species followed the classification proposed by the “Angiosperm Phylogeny Group” [61]. Galls were recorded only once at each collection point for estimating richness. The galls were photographed and categorized according to color, shape presence or absence of trichomes and the organ on which they occurred. The identification of galling insects was done whenever possible.

### Data analysis

In order to determine whether local and region species richness are correlated, linear regression analyses were used, where the y variable was the mean of the richness of galling insects and host plants in the 10 sampling points within each mountain; the x variable was the regional species richness—sum of the species on each mountain. In this way, the degrees of freedom were the number of mountains minus one, thus avoiding pseudoreplication by considering the collection points within each mountain as independent points [62]. The analyses were followed by the inspection of the residuals to verify the fulfillment of the assumptions of the model.

For one part of this work, the additive separation of diversity levels was adopted, according to the proposition of [26]: gamma = alpha + beta. Gamma diversity is considered the total number of species of each mountain, while alpha diversity is the average number of species form the 10 sampling points within each mountain. In this way, beta diversity is obtained by the subtraction of alpha diversity from gamma diversity, rather than being obtained by division. The approach of additive separation of diversity into its components α and β allows them both to be expressed in the same units and allows a direct evaluation of the relative contribution of each to regional diversity, and was adopted only to facilitate the graphic analysis of these components [27,63,64]. We also assessed what generated the beta diversity pattern using a deconstruction approach in the package betapart [29]. In this step, the multiplicative beta (i.e., gamma = alpha * beta) was adopted.e According to [28], the use of the multiplicative model is mandatory because the independence between alpha and beta is a necessary assumption. Betapart provides a unified framework for the partitioning of total dissimilarity into the components of spatial turnover and nestedness. Thus, β was deconstructed into two components, the Sørensen (β_SOR_) and Simpson (β_SIM_) indices. The β_SIM_ represents spatial species turnover whereas β_SNE_ (obtained by the difference between β_SOR_ and β_SIM_) shows the loss or gain of species due to nestedness [28]. To examine the contribution of each sampling level to total biodiversity an additive partitioning of the data was performed with four spatial scales degrees of diversity: (α1) diversity within plots, (β1) diversity between plots, (α2) diversity within mountains (β2) diversity between mountains, and (α3) diversity within ranges (i.e., Mantiqueira and Espinhaço) and (β3) diversity between ranges. The same routine was applied for the mountains of the Mantiqueira and Espinhaço ranges separately. Multiple-site dissimilarity was computed 1000 times for randomly sampled subsets of 10 sites (command beta.sample in R package betapart), and the resulting distributions of βSIM and βSNE values across the 1000 samples were used to empirically assess whether there were significant differences. Observed and expected diversities for α and β components were considered significantly different when p<0.05. All of the analyses were performed in the statistical R package [65].

## Results

In the universe of 11,000 individual plants collected from 110 sampling points distributed among the 11 mountains, 382 species of galling insects were recorded (316 from Espinhaço, and 101 from Mantiqueira Range), and 537 species of plants (421 from Espinhaço, and 167 from Mantiqueira Range) S1 File.

**Fig 3:**
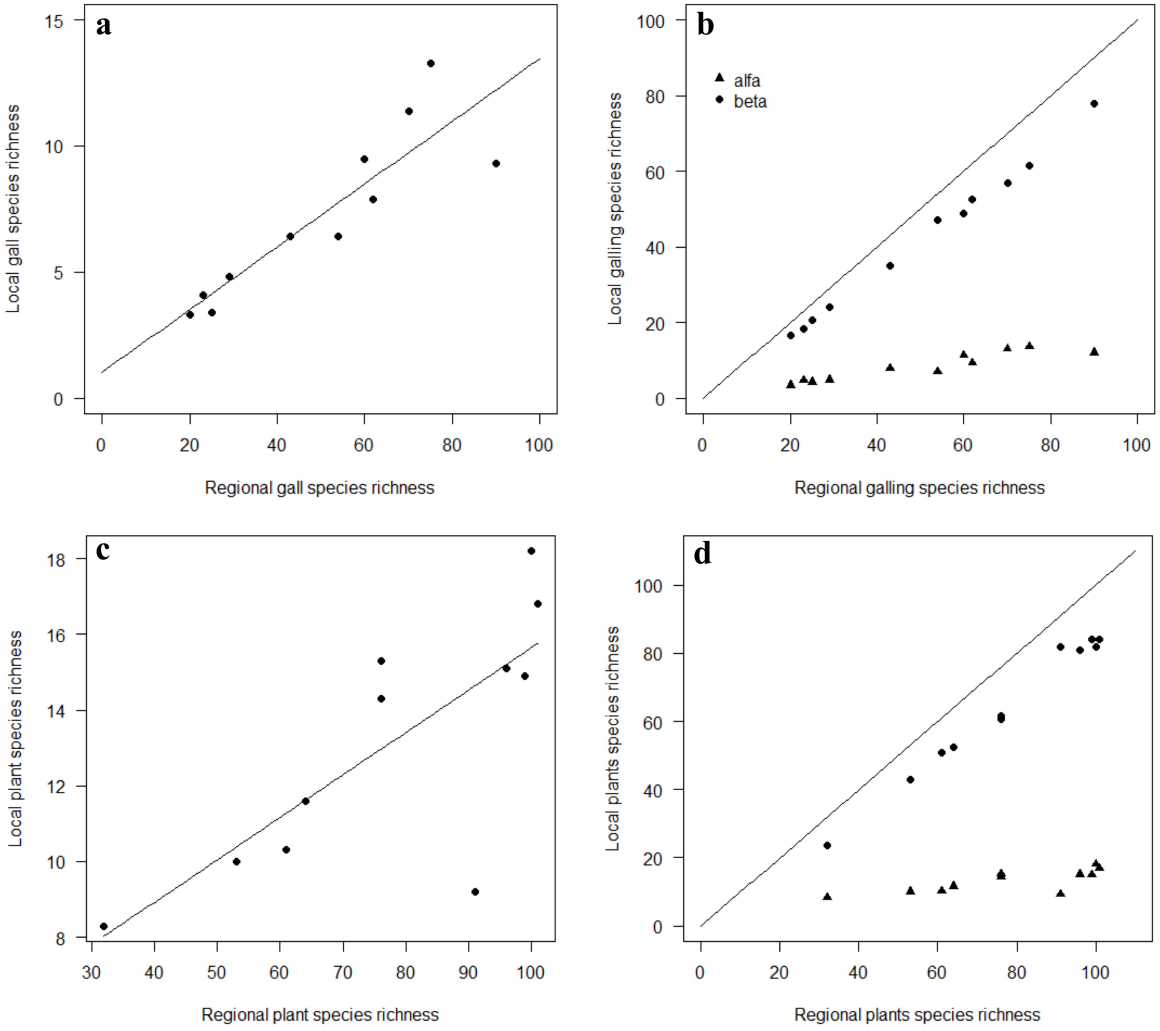
a. Relationship between local and regional richness of galling insects (R^2^ = 0.78; F_1,10_ = 32.45; p<0.01); b. Relationship between regional and local richness of galling insects. The local richness, or α (**▲**), of galling insects in relation to regional richness. The richness β (•), which incorporates most of the regional richness. The diagonal line represents the theoretical limit (local richness = regional richness). c. The relationship between local and regional richness of host pants (R^2^ = 0.58; F_1,10_ = 12.73; p<0.01); d. Relationship between regional richness and local richness of host plants. The local richness, or α (**▲**), of host plants in relation to regional richness. The richness β (•), which incorporates most of the regional richness. The diagonal line represents the theoretical limit (local richness = regional richness).

The local species richness of galling insects and host plants increased with regional richness of species, suggesting a pattern of unsaturated communities (Galls: R^2^ = 0.75; F_1,10_ = 32.45; p<0.01; Fig 3a; Plants: R^2^ = 0.54; F_1,10_ = 12.73; p<0.01; Fig 3c). The variation in local richness is explained by the increase in regional richness for galling insects and plants, at 54% and 75%, respectively.

**Fig 4:**
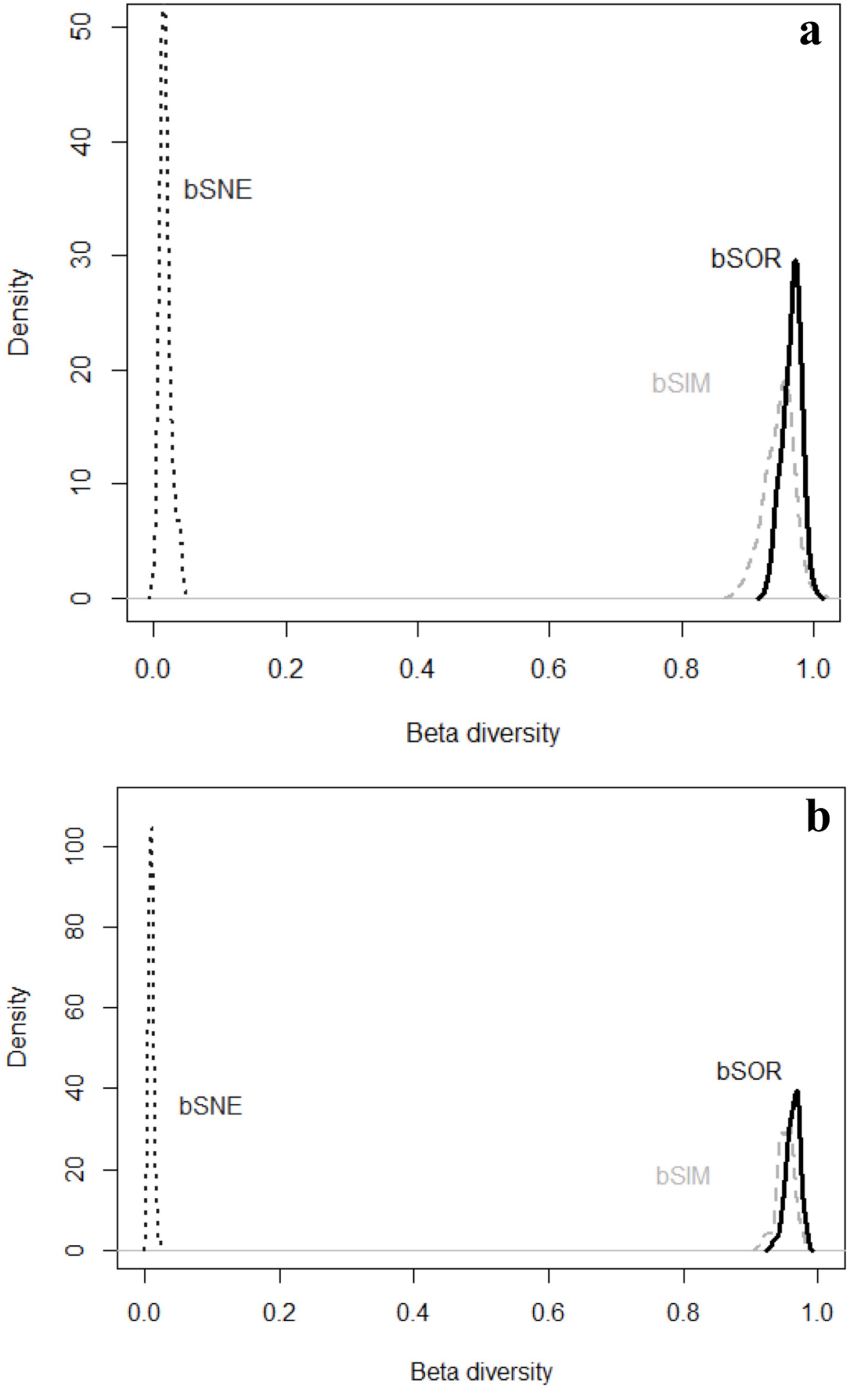
Density plots representing the distribution of the β_SOR_ (solid line) into β_SIM_ (dashed grey line) and β_SNE_ (dashed black line) across 1000 samples of 10 sites from each data set: a. galling insects, b. plants.

The additive partition of regional richness (γ) into its local and beta components showed that local richness (α) of species of galling insects and host plants are low relative to regional richness; the beta component (β) incorporates most of the regional richness (Fig 3b and 3d). The beta component incorporated 96.48% for βSOR_GALLS_, and 95.03% for βSOR_PLANTS_, of the regional richness of gall inducing insects and host plants, while the local component (α) of gall inducing insects and host plant richness incorporated 3.52% for α_GALLS_, and 4.97% for α_PLANTS_, respectively, of regional richness (Fig 4).

The multi-scale analysis of additive partitioning showed similar patterns for galling insects and host plants. The alpha components at the plot scale— α1_GALLS_ = 2.2%, α1_PLANTAS_ = 2.43%—were significantly higher than expected. The alpha components at the mountain scale— α2_GALLS_ = 13.27%, α2_PLANTAS_ = 13.52%—and the scale of the mountain range— α3_GALLS_ = 54.84%, α3_PLANTAS_ = 54.84%—were significantly lower than expected. While the beta components at the plot scale— β1_GALLS_ = 11.06%, β1_PLANTAS_ = 11.01%—at the mountain scale— β2_GALLS_ = 41.56%, β2_PLANTAS_ = 41.31%—and at the mountain range scale— β3_GALLS_ = 45.15%, β3_PLANTAS_: = 45.15%—were significantly lower than expected.

**Fig 5:**
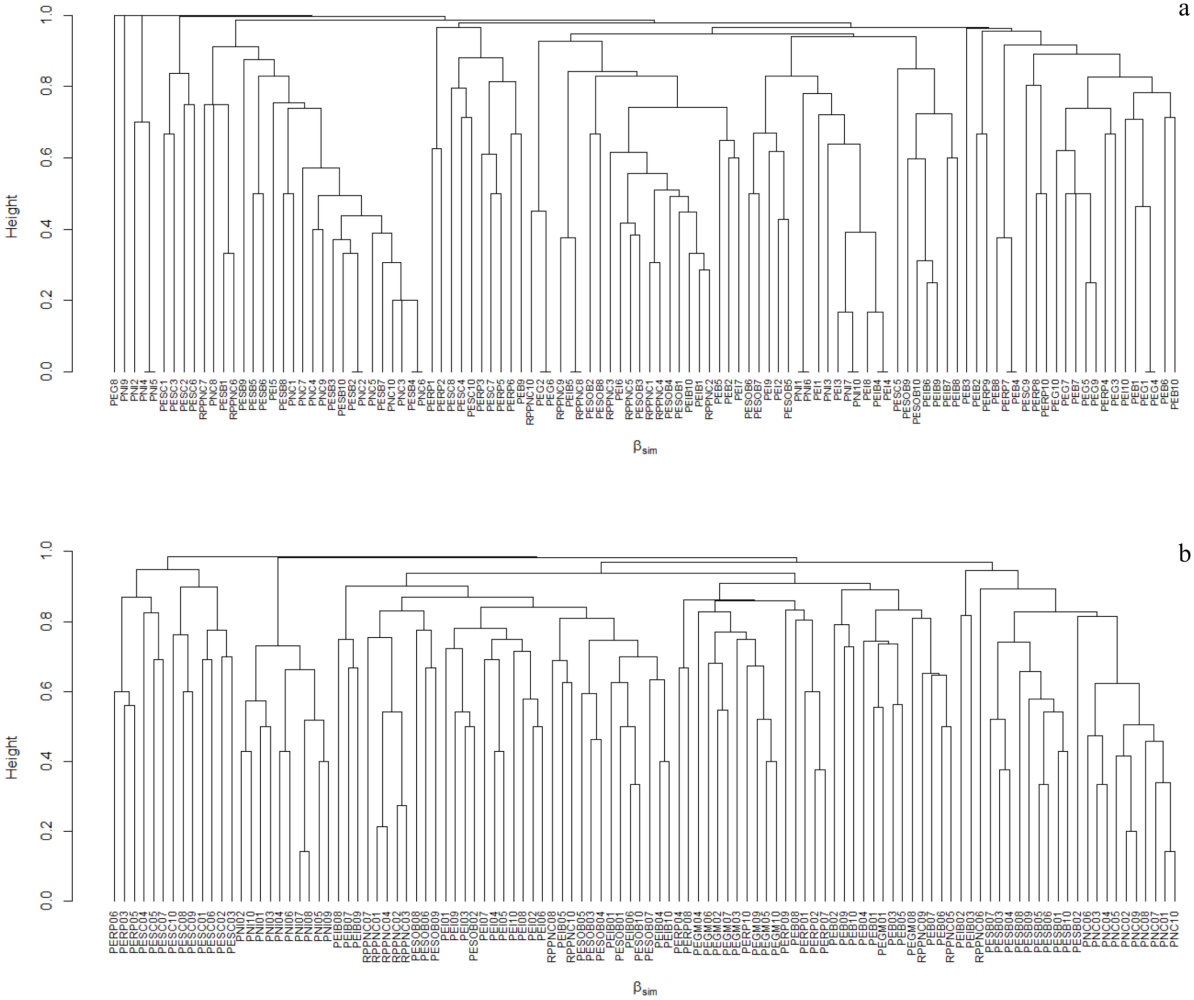
Clustering using the average linkage of the bsimpson components for insect galls (Claster “a”) and for host plants (cluster “b”) between sample plots located on eleven mountains—10 samples from each mountain.

**Fig 6:**
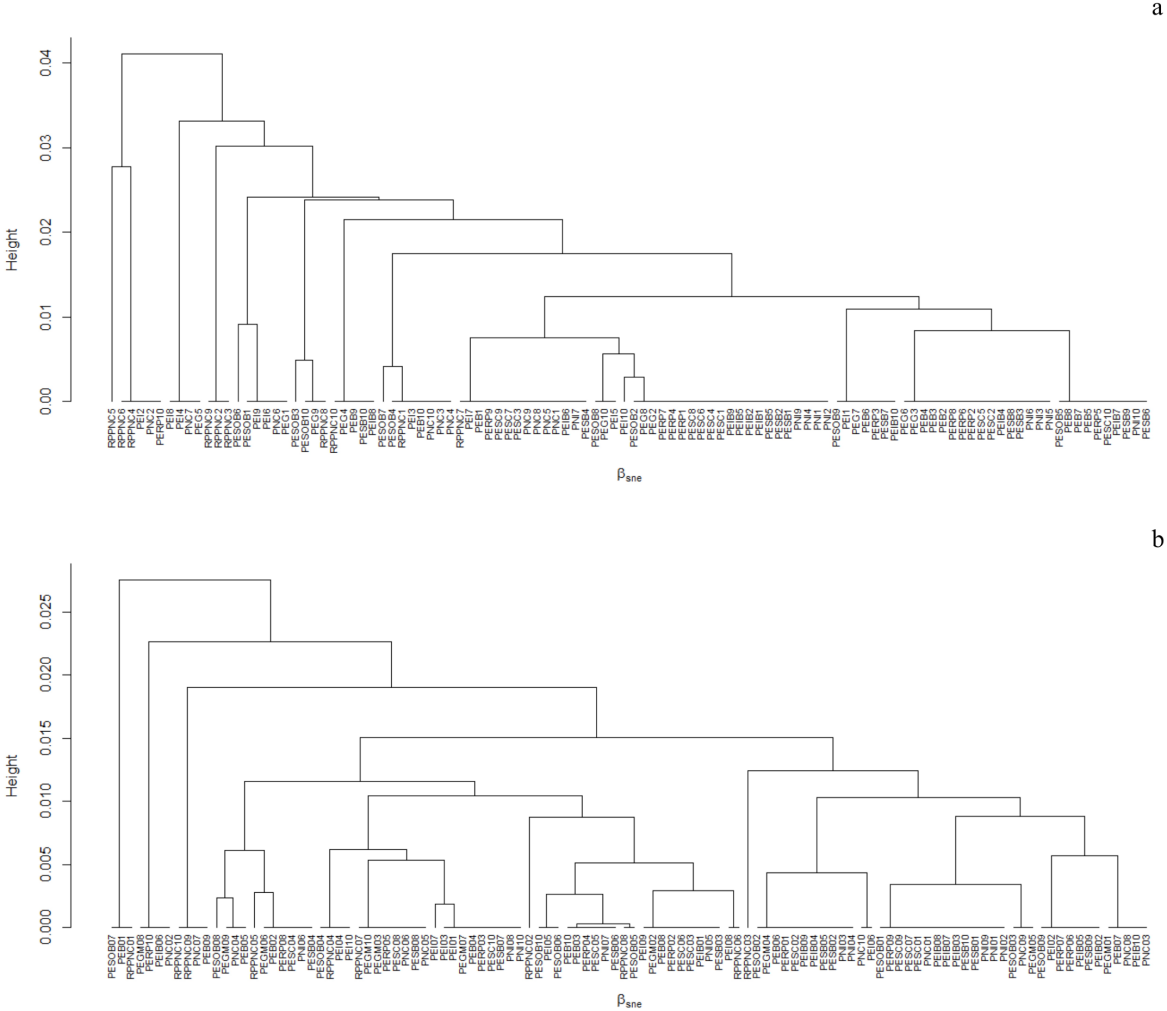
Clustering using the average linkage of the bsne components for insect galls (Claster “a”) and for host plants (cluster “b”) between sample plots located on eleven mountains—10 samples from each mountain.

The beta diversity of galling insects and host plants— βSOR_GALLS_ = 96.48%, βSOR_PLANTS_ = 95.03% was mainly due to the process of turnover—βSIM_GALLS_ = 94.69%, βSIM_PLANTS_ = 93.67%—and minimally by nesting— βSNE_GALLS_ = 1.88%, βNES_PLANTS_ = 1.35% (Figs 5 and 6). When comparing the two mountain ranges, Espinhaço and Mantiqueira, we saw that the beta diversity for both galling insects— βSOR_ESPINHAÇO_ = 95.24%, βSOR_MANTIQUEIRA_ = 93.22%—and host plants— βSOR_ESPINHAÇO_ = 93.24%, βSOR_MANTIQUEIRA_ = 90.52%—was primarily due to the process of turnover in both mountain ranges—βSIM_GALLS-ESPINHAÇO_ = 93.41%, βSIM_GALLS-MANTIQUEIRA_ = 88.96%; βSIM_PLANTS-ESPINHAÇO_ = 91.66%, βSIM_PLANTS-MANTIQUEIRA_ = 88.27%—and a minority by nesting— βNES_GALLS_-_ESPINHAÇO_ = 1.82%, βSNE_GALLS-MANTIQUEIRA:_ = 4.25%, βSNE_PLANTS_-_ESPINHAÇO_ = 1.57%, βSNE_PLANTS_-_MANTIQUEIRA_ = 2.25%.

## Discussion

The local richness of galling insects was positively correlated with regional richness, suggesting that mountains richer in galling insect species have local habitats with more species of galling insects (Fig 3a). The same pattern was found for plants (Fig 3c). This unsaturation pattern suggests that local richness does not reflect the biotic interactions, especially antagonistic, that occur in the habitat, and are being generated by non-interactive processes [22,34,35]. This pattern of community unsaturation (i.e., local richness being a proportional sample of its regional richness) is a reflection of the high specificity and distribution of the host plants (high values of β richness) and is commonly found in communities of herbivorous insects, and especially galling insects [30,32]. Many critics have emerged around the interpretation of the correlation between local and regional diversity face a scale dependence. For [63], local diversity could be seriously constrained by the scale. The physical constrains of small scales could drive local and regional curves to a saturated pattern, but in this case, not in consequence of biotic interactions. The sample design of this dataset can be considered small. Despite that we have found evidences of an unsaturated patterns for both, galling insects and plants, pointing to no interactive mechanisms. The conclusions of this clear pattern must be view with caution and the local and regional richness should not be analyzed independently of beta diversity and it process under a multiple scale approach—turnover and nestedness—as we intended in the followed discussion.

Despite the problems with a lack of standardization of samples in previous works, the evidence accumulated so far suggests that the distribution of galling insects has low similarity among sampling sites [22,30,56,58,66,67]. The results of this work corroborate the evidence discussed thus far in the literature. The additive partition of regional richness (γ) into its local and beta components shows that the local richness (α) of galling insect species and host plants are low relative to regional richness, and that the beta (β) component incorporates most of the regional richness (Fig 3b and 3d). In addition, the multi-scale analysis of the additive partitioning of the diversity showed similar patterns for galling insects and host plants for the scales of plot, mountain and mountain range, with the beta component contributing the most to regional diversity at all scales.

The beta diversity for galling insects and host plants mainly reflect the process of turnover, and a minority of nesting, both when the whole of the 11 mountains were analyzed and when the two mountain ranges were analyzed separately—Mantiqueira and Espinhaço Ranges; although the nesting process explained more of the beta diversity of galling insects and plants located in the Mantiqueira Range (Figs 5 and 6). The set of results show that in altitudinal fields and rupestrian grasslands there are abrupt changes in the floristic composition between habitats along the altitudinal gradient. These abrupt changes may reflect geographical isolation due to barriers imposed by relief, topography and soil patches [22]. The Mantiqueira and Espinhaço are mountain ranges with distinct geological origins, however, they share common ecological patterns and processes and retain some floristic similarity [68]. For example, applying the Sørensen similarity index to species lists extracted from 31 studies conducted in rupestrian grasslands and altitudinal fields, [68] concluded that at the genus level it is not possible to distinguish floristically these two physiognomies. Altitudinal fields are formed of shrub vegetation and slow-growing low trees immersed in a grassy matrix. This vegetation is closely controlled by topography, drainage systems and the distribution of soil types. In general, the soils of altitudinal fields possess greater physico-chemical similarities with paramo-andean soils [42,43], particularly those described from similar geological substrates (i.e., plutonic and high grade metamorphic rocks) than with rupestrian grasslands [42,43]. Although there is significant horizontal and vertical variation, the soils of the altitudinal fields are typically humic and dark in the upper horizons and redish-yellow, clayey, sometimes podzolized, acidic and moderately fertile in lower horizons [46]. In addition, altitudinal fields are immersed in the phytogeograhical domain of the Atlantic Forest, therefore tree lines isolate them from the ombrophylous forests located in lower altitudes by abrupt ecotones [46]. Rupestrian grasslands are formed by a mosaic of vegetation associated with distinct soil patches with different physical and chemical characteristics. Studying arbuscular mycorrhizal fungi, [69] identified five distinct habitats associated with rupestrian grasslands—e.g., rocky outcrops, stony and sandy fields, sandy marshes and peat bogs—and have demonstrated that physical attributes, such as the soil granulometric composition, are more relevant than the chemical attributes in explaining this important and fundamental ecological interaction, and are the attributes of the soil that vary among habitats [70,71]. [72] has already demonstrated that the vegetative mosaic of rupestrian grasslands, despite sharing a stress-tolerant flora, have plant communities with different functional attributes associated with specific soil conditions. In addition, rupestrian grasslands are predominantly immersed in the phytogeographical domain of the Cerrado, with which they retain greater ecological similarity, such as their climatic regime and resistance to fire [50]. Fire has been analyzed as an important evolutionary factor for the process of speciation and irradiation of galling insects in the Cerrado and rupestrian grasslands because it causes frequent and synchronous regrowth of vegetation [15,73]. This may be, along with plant richness, one of the most important processes that direct the differences in regional richness of galling insects between rupestrian grasslands and altitudinal fields, although fire also plays an important role, albeit to a lesser extent, in vegetation of altitudinal fields [48]. Therefore, as with altitudinal fields, the high diversity of vegetation of rupestrian grasslands has been attributed to the mosaic of environments formed by various soil classes, rugged relief and microclimatic variation [50,69]. These mosaics are added to the geographic barriers imposed by the relief. In rupestrian grasslands, populations are generally disjointed, often restricted to small geographically isolated mountains [74]. As a result, there are a large number of species and high endemism of the plants and, consequently, a large discontinuity in the composition of plant species and their associated herbivores. The mosaic of local habitats showed the strongest force behind the high beta diversity recorded in plant-galling interactions.

Although the composition of galling insect and host plant species varies among the sample sites, mountains and even mountain ranges, local richness remains relatively low. In this way, the addition of local habitats with different landscapes substantially affects the regional richness of galls, as well as the vegetal community, at the mountain scale, and their conservation can be designed to include small landscapes with different characteristics instead of a single large and homogeneous landscape. The mountain top grasslands are not composed by a continuum but a mosaic of habitats what points to a predominance of environmental constrains, instead interactive process, as the main force driving the special distribution of rupestrian grasslands diversity. In addition, the data showed that, for both the Mantiqueira and Espinhaço Ranges, each mountain contributes in a fundamental way to the composition of regional diversity of galling insects and host plants. Therefore, the design of future conservation strategies should incorporate multiple spatial scales.

## Supporting information

S1 File List of plants, richness of plants and coordinates of the sample points. (XLSX)
